# Supplementary figures and images for: Targeted-Gene Sequencing to Catch Triple Negative Breast Cancer Heterogeneity before and after Neoadjuvant Chemotherapy
Source: Cancers (Basel). 2019 Nov 8;11(11):1753. doi: 10.3390/cancers11111753 (PMC6895966; doi:10.3390/cancers11111753)

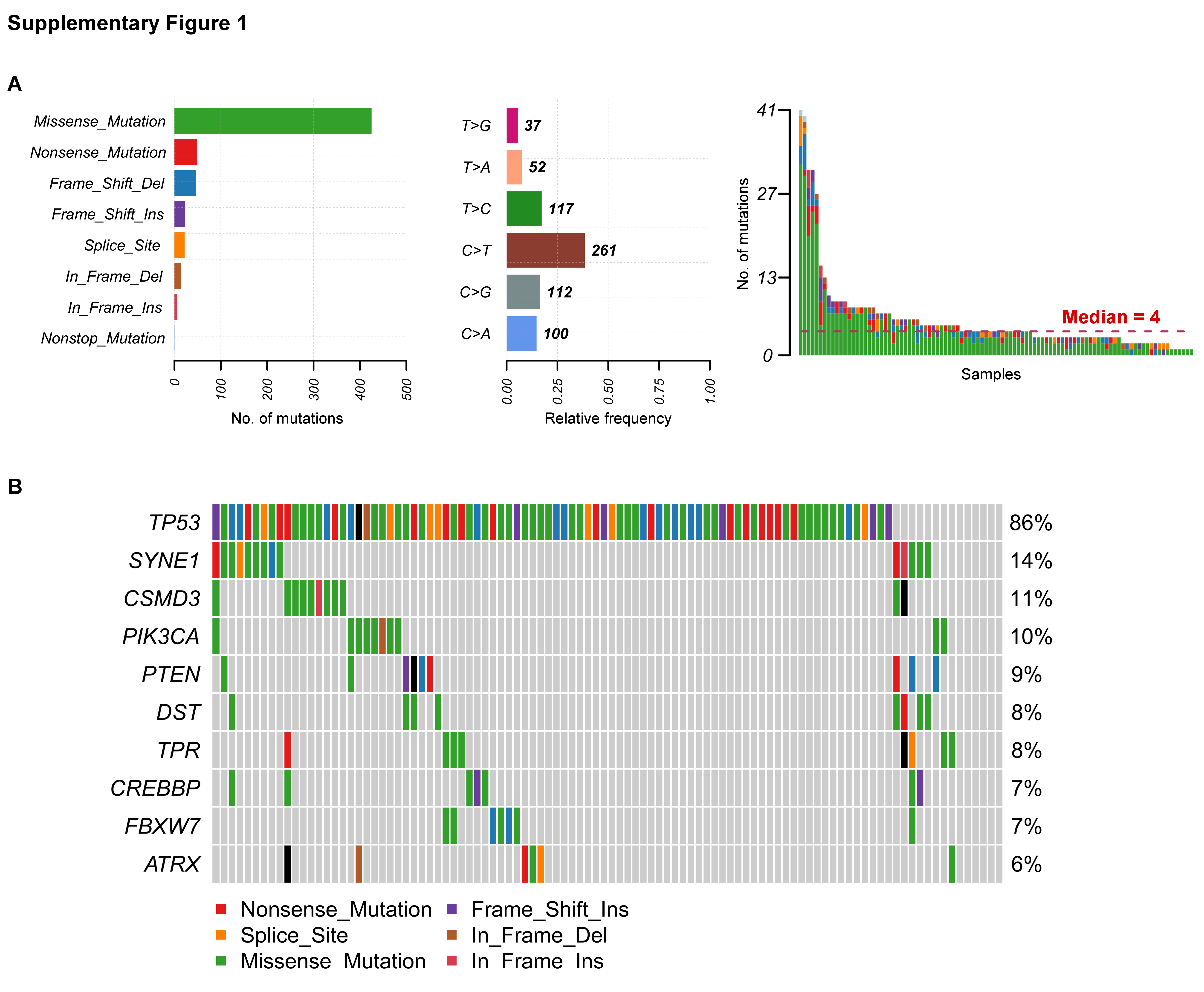

Supplement: Supplementary file 1 [file cancers-11-01753-s001.zip › SuppFigure1.tif]

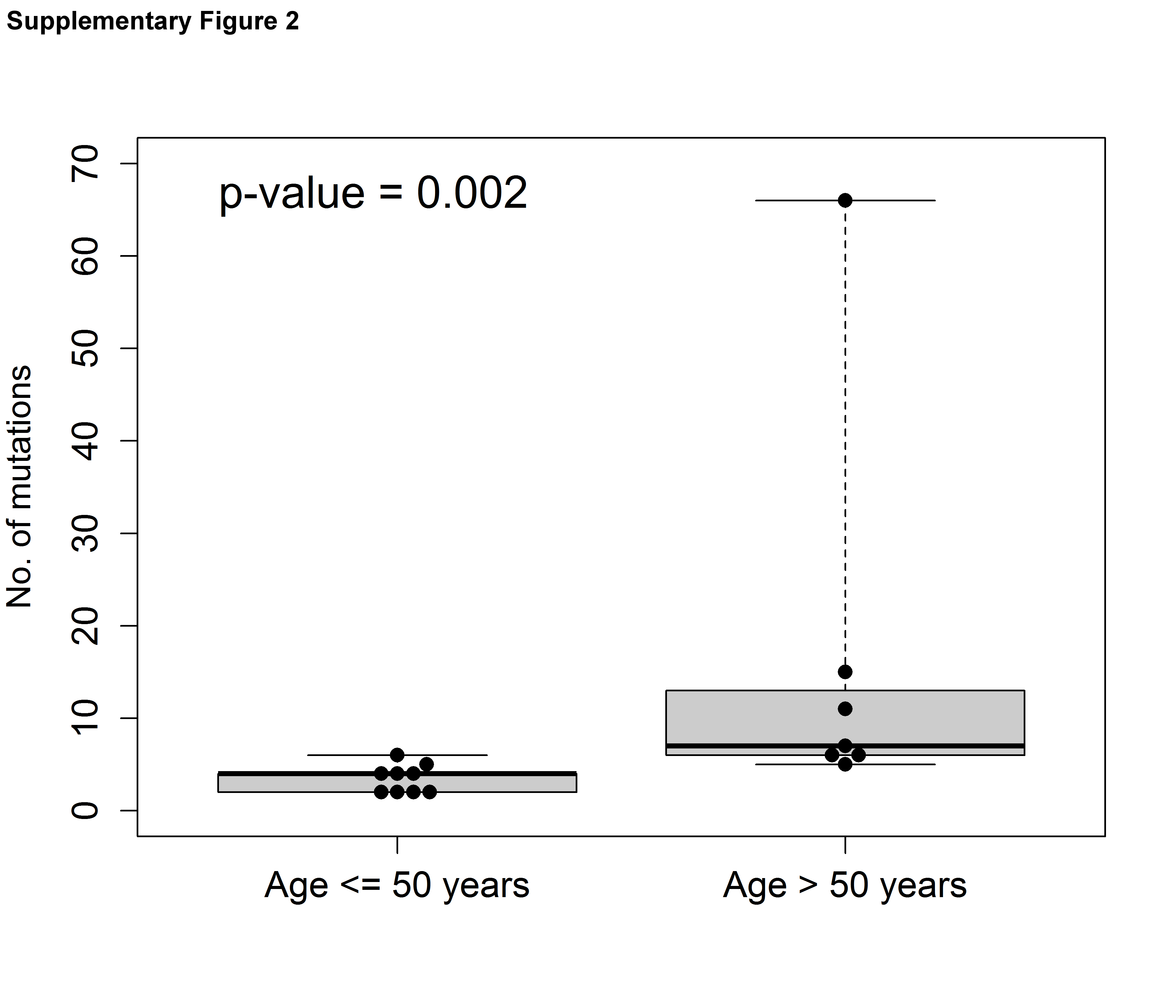

Supplement: Supplementary file 1 [file cancers-11-01753-s001.zip › SuppFigure2.tif]

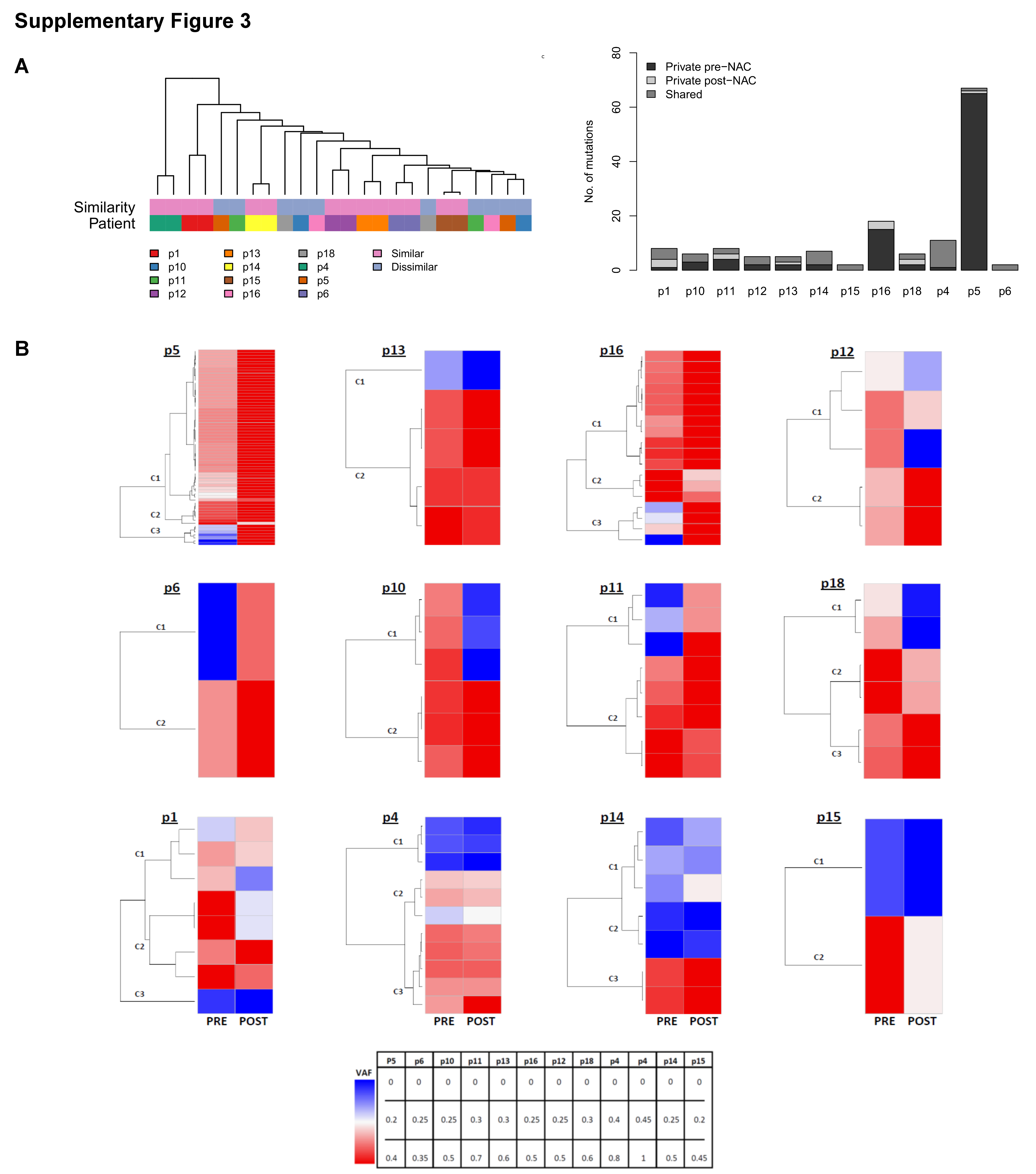

Supplement: Supplementary file 1 [file cancers-11-01753-s001.zip › SuppFigure3.tif]
